# Histone deacetylase 6 inhibition improves memory and reduces total tau levels in a mouse model of tau deposition

**DOI:** 10.1186/alzrt241

**Published:** 2014-02-27

**Authors:** Maj-Linda Selenica, Leif Benner, Steven B Housley, Barbara Manchec, Daniel C Lee, Kevin R Nash, Jay Kalin, Joel A Bergman, Alan Kozikowski, Marcia N Gordon, Dave Morgan

**Affiliations:** 1 Byrd Alzheimer’s Institute, University of South Florida, Tampa, FL 33613, USA; 2 College of Pharmacy, Department of Pharmaceutical Sciences, University of South Florida, Tampa, FL 33612, USA; 3 College of Medicine, Department of Molecular Pharmacology and Physiology, University of South Florida, Tampa, FL 33612, USA; 4 Department of Pharmacology and Molecular Science, The Johns Hopkins University, School of Medicine, Baltimore, MD 21205, USA; 5 Drug Discovery Program, Department of Medicinal Chemistry and Pharmacognosy, College of Pharmacy, University of Illinois at Chicago, Chicago, IL 60612, USA

## Abstract

**Introduction:**

Tau pathology is associated with a number of age-related neurodegenerative disorders. Few treatments have been demonstrated to diminish the impact of tau pathology in mouse models and none are yet effective in humans. Histone deacetylase 6 (HDAC6) is an enzyme that removes acetyl groups from cytoplasmic proteins, rather than nuclear histones. Its substrates include tubulin, heat shock protein 90 and cortactin. Tubastatin A is a selective inhibitor of HDAC6. Modification of tau pathology by specific inhibition of HDAC6 presents a potential therapeutic approach in tauopathy.

**Methods:**

We treated rTg4510 mouse models of tau deposition and non-transgenic mice with tubastatin (25 mg/kg) or saline (0.9%) from 5 to 7 months of age. Cognitive behavior analysis, histology and biochemical analysis were applied to access the effect of tubastatin on memory, tau pathology and neurodegeneration (hippocampal volume).

**Results:**

We present data showing that tubastatin restored memory function in rTg4510 mice and reversed a hyperactivity phenotype. We further found that tubastatin reduced the levels of total tau, both histologically and by western analysis. Reduction in total tau levels was positively correlated with memory improvement in these mice. However, there was no impact on phosphorylated forms of tau, either by histology or western analysis, nor was there an impact on silver positive inclusions histologically.

**Conclusion:**

Potential mechanisms by which HDAC6 inhibitors might benefit the rTg4510 mouse include stabilization of microtubules secondary to increased tubulin acetylation, increased degradation of tau secondary to increased acetylation of HSP90 or both. These data support the use of HDAC6 inhibitors as potential therapeutic agents against tau pathology.

## Introduction

Tauopathies are neurodegenerative disorders for which there are no effective treatments. Some disorders are caused by mutations in tau that increase the probability of tau aggregate formation, leading to intracellular neurofibrillary tangles [1]. These disorders are typically referred to as fronto-temporal dementias. Other tauopathies occur in different brain regions (corticobasal syndrome, progressive supranuclear palsy, and so forth) [2]. The most common disorder demonstrating tau inclusions is Alzheimer’s disease, where the tau pathology correlates better than amyloid pathology with cognitive impairment [3].

Histone deacetylases (HDACs) are a family of proteins that remove acetyl moieties attached covalently to lysine residues in proteins. In the cell nucleus, HDACs catalyze the deacetylation of histones and, in general, promote chromatin condensation and repression of gene expression. In transformed cells, these enzymes are thought to suppress proapoptotic programs, leading to unregulated proliferation. As such, HDAC inhibitors are widely explored as treatments for cancer [4]. There are at least 18 isoenzymes in the HDAC family, divided into four homology classes. Classes I, II and IV are zinc dependent, while class III, also known as sirtuins, are NAD^+^ dependent for their enzyme activity. Class I HDACs (HDAC1, HDAC2, HDAC3 and HDAC8) are nuclear enzymes and are the major focus of research for anti-tumor agents. Class II enzymes are often tissue specific, divided into class IIa enzymes (HDAC4, HDAC5, HDAC7, HDAC9) that shuttle between cytoplasmic and nuclear compartments and class IIb enzymes (HDAC6 and HDAC10) that are primarily cytoplasmic and deacetylate nonhistone proteins. HDAC6 has been shown to act upon tubulin, cortactin and HSP90. Tubulin acetylation is associated with increased microtubule stabilization [5].

The Kozikowski laboratory has synthesized a number of compounds focusing on HDAC6. One such agent is tubastatin A (tubastatin). This molecule was found to have nanomolar potency in inhibiting HDAC6, but requires micromolar or greater concentrations to inhibit most other HDACs (>1,000× selectivity for all but HDAC8, at 50-fold selectivity). This agent was found to increase the acetylation of tubulin in cells, but not histone H4 proteins. Moreover, tubastatin treatment was found to be protective against homocysteic acid-induced oxidative stress [6]. This agent reduced the phenotype in a model of Charcot-Marie-Tooth disease [7]. This disorder is caused by mutations in the 27 kDa small heat shock protein HSBP1, leading to decreased tubulin acetylation and axonal atrophy. Tubastatin treatment prevented both the loss of acetylated tubulin and axonopathy. Recent observations also demonstrate that decreases in HDAC6 activity or expression promote tau clearance [8], while HDAC6 mutations rescued tau-induced microtubule defects in a Drosophila model of tau pathology [9]. Given the observation that phosphorylation of tau results in dissociation from tubulin and decreased stabilization of microtubules [10], we hypothesized that stabilizing microtubules by acetylation might counteract the phenotype found in the rTg4510 mouse model of tauopathy. Moreover, we chose to start injecting mice at an age when there was already memory loss and considerable tau pathology because, even at the earliest stages, Alzheimer patients have substantial existing tau deposition [11]. We have thus treated 5-month-old rTg4510 mice with the HDAC6 selective drug tubastatin for 2 months, and monitored its effect on the behavioral and pathological phenotype of this mouse.

## Materials and methods

### Drug preparation

Tubastatin was synthesized and provided by Dr A Kozikowski [6] (University of Illinois at Chicago, Chicago, IL, USA). The compound was dissolved in 0.9% saline to reach a dose of 25 mg/kg body weight per animal. This dose has been shown to efficiently inhibit HDAC6 *in vivo*[7]. The solution was sonicated in the water bath for 5 minutes at room temperature until is fully solubilized.

### Animals and injection procedure

The production of rTg4510 mice, bred from parental P301L tau and tTA lines, was maintained as originally described by Santacruz and colleagues [12]. We administered experimental agents using daily intraperitoneal injections into rTg4510 mice and nontransgenic littermates from 5 to 7 months of age. Each group consisted of six animals per genotype. Mice were weighed, and injected with tubastatin (25 mg/kg) or 0.9% normal saline solution (vehicle). Animal procedures were consistent with the recommendations of the National Research Council’s *Guide for the Care and Use of Laboratory Animals* and were approved by the University of South Florida Animal Care and Use Committee (IACUC).

### Behavior methods

Behavioral analysis was carried out as described previously [13-17]. For 3 days before testing, mice were weighed and handled in order to habituate the mice to contact with the experimenter. The sequence of tests over a 2-week period were Y-maze, open field, rotarod, radial arm water maze, water maze reversal, open pool, and fear conditioning.

#### Y-maze

Each animal was placed in a Y-maze for a single 5-minute trial, during which the sequence and total number of arm choices were recorded. Spontaneous alternation, expressed as a percentage of total alternations, was calculated as described [18]. If an animal made the following sequence of arm selections (1,2,3,2,1,3,1,2), the total alternation opportunities will be six (total entries minus two) and the percentage alternation would be 67% (four out of six). Chance performance is 50%

#### Open field

The open field is used as a standard test of general activity. Animals were monitored for 15 minutes in a 40 cm square open field with video tracking software, under moderate lighting. General activity levels were evaluated by measurements of horizontal activity and vertical activity. The open field test can also be used to measure anxiety levels, as assessed by the ratio of center distance traveled to total distance traveled. As the natural tendency of the animal is to avoid open areas, animals that show greater tendency to explore in the center of the field show a lower tendency for anxiety, or the reverse situation can indicate a higher anxiety level.

#### Rotarod

Motor performance was assessed by placing the mice onto the round portion of a circular rod that was then accelerated slowly. Mice need to walk at the speed of rod rotation to keep from falling. The time until falling was recorded for each mouse. Mice were given four trials each day for 2 consecutive days.

#### Two-day radial arm water maze

The 2-day radial arm water maze has been previously described in detail [19]. Briefly, the radial arm water maze consists of a 1 m pool with six swim paths (arms) radiating out of an open central area, with a hidden escape platform located at the end of one of the arms. On each trial, the mouse was allowed to swim in the pool for up to 60 seconds to find the escape platform. Incorrect arm entries or failure to select an arm for 15 seconds were counted as errors. For a given mouse, the platform was located in the same arm on each trial, but the start arms were varied for each trial so that mice rely upon spatial cues to solve the task instead of learning motor rules (that is, second arm on the right). On day 1, mice were given 15 trials where each block consist of three trials (five blocks in total) alternating between a visible platform (above the water) and a hidden platform (below the water). The following day, mice were given 15 additional trials (five blocks), all using a hidden platform. The goal arm location for sequential mice was different to avoid odor cues from revealing the goal arm.

#### Radial arm water maze reversal

This test was completed on the day after the 2-day radial arm water maze and was performed under the same conditions. The only difference was that the goal arm was located 180º from the original location. This reversal task tests the ability of the mice to extinguish a learned memory to create a novel one. Fifteen trials were given in a single day, all with a hidden platform.

#### Visible platform

This was conducted in an open pool (no swim arms) to ensure mice can see and perform a platform task [20]. The visible platform was placed 0.8 cm above the water surface and had an attached 10 cm × 40 cm ensign. For the test day, the animal was placed into the pool facing the wall in each of the four quadrants of the pool. Latency to find and ascend the visible platform was recorded (maximum 60 seconds).

#### Associative fear conditioning

Fear conditioning was used to access both contextual and cued memory formation. For these experiments, an aversive stimulus (in this case, a mild foot shock, 0.5 mA) was paired with an auditory conditioned stimulus (white noise) within a novel environment. These tests were given as follows: animals were placed in the fear conditioning apparatus for 2.5 minutes, then a 30-second acoustic conditioned stimulus (white noise, 70 dB) was coupled with a 0.5 mA shock (unconditioned stimulus) applied to the floor grid during the last 2 seconds of the conditioned stimulus. Two minutes later the conditioned stimulus and unconditioned stimulus were again administered, and the mice remained in the conditioning chamber for an additional 2 minutes. The mice were placed in the same chamber and monitored for freezing to the context 24 hours later (no shocks or auditory cue given). Immediately after the contextual test, mice were placed into a novel context and exposed to the conditioned stimulus for 3 minutes (cued fear conditioning). Both the contextual and the cued recall tests were performed the next day ~24 hours following training. Learning was assessed by measuring freezing behavior (that is, motionless position).

### Tissue collection

Two months post intraperitoneal injections, mice were weighed, overdosed with pentobarbital (200 mg/kg) and perfused with 25 ml of 0.9% normal saline solution. Mice were placed on an isothermal pad after anesthesia and during perfusion to avoid artifactual tau phosphorylation caused by reductions in body temperature [21]. Brains were collected from the animals 1 hour after the last tubastatin injection, following saline perfusion, and were hemisected down the sagittal midline. One hemisphere was dissected and frozen with dry ice for biochemical studies. The second hemisphere was immersion fixed in 4% paraformaldehyde for 24 hours, and cryoprotected in successive incubations of 10%, 20% and 30% solutions of sucrose for 24 hours in each solution. Subsequently, the fixed hemispheres were frozen on a cold stage and sectioned in the horizontal plane (25 μm thickness) on a sliding microtome and stored in Dulbecco’s phosphate-buffered saline with 10 mM sodium azide solution at 4°C.

### Immunohistochemical/histological procedure and analysis

Eight sections, 200 μm apart, were chosen for analysis using immunohistochemical procedural methods described previously [22]*.* For each marker, floating sections from all animals were placed in multi-sample staining trays, and endogenous peroxidase was blocked (10% methanol, 10% H_2_O_2_ in phosphate-buffered saline; 30 minutes). Tissue samples were permeabilized (with 0.2% lysine, 1% Triton X-100 in phosphate-buffered saline solution), and incubated overnight in appropriate primary antibody (clonal names in parentheses). Anti-N-terminus of human tau antibodies (H150; Santa Cruz Biotechnology, Dallas, Texas, USA), anti-phospho-tau at serine 396 antibodies (pSer396; Anaspec, Fremont, CA, USA), anti-phospho-tau at serine 199/202 antibodies (pSer199/202; Anaspec, Fremont, CA, US), and anti-phospho-tau at serine 202 and threonine 205 antibodies (biotinylated AT8; ThermoScientific, Waltham, MA, USA) were used in this study. Sections were washed in phosphate-buffered saline and then incubated in corresponding biotinylated secondary antibody, except for AT8 (pSer199/Thr205; Vector Laboratories, Burlingame, CA, USA). The tissue was again washed after 2 hours, and incubated with the Vectastain® Elite® ABC kit (Vector Laboratories, Burlingame, CA, USA) for enzyme conjugation. Finally, sections were developed using 0.05% diaminobenzidine, 0.5% Ni^2+^ and 0.03% H_2_O_2_. Tissue sections were then mounted onto slides, dehydrated, and cover slipped. Each immunochemical assay omitted some sections from primary antibody incubation to evaluate nonspecific reaction of the secondary antibody. Prior work with nontransgenic mice confirmed the specificity of the anti-tau antibodies for tau in rTg4510 mice.

Gallyas histology was performed as described previously [23], using sections that were premounted on slides and then air-dried for a minimum of 24 hours. The sections were rehydrated for 30 seconds prior to proceeding with the Gallyas protocol. Slides were treated with 5% periodic acid for 5 minutes, washed with water, and incubated sequentially in silver iodide (1 minute) and 0.5% acetic acid (10 minutes) solutions prior to being placed in developer solution (2.5% sodium carbonate, 0.1% ammonium nitrate, 0.1% silver nitrate, 1% tungstosilicic acid, 0.7% formaldehyde). Slides were treated with 0.5% acetic acid to stop the reaction, incubated with 0.1% gold chloride, placed in 1% sodium thiosulfate, and counterstained with 0.1% nuclear fast red in 2.5% aqueous aluminum sulfate, with each step separated by washes in water. Following a final wash, slides were dehydrated and cover slipped.

Stained sections were imaged using a Zeiss Mirax150 digital scanning microscope (Carl Zeiss MicroImaging GmbH Clinical 07740 Jena, Germany). The area of positive stain in each hippocampal section was analyzed. The software used hue, saturation and intensity to segment the image fields. Thresholds for object segmentation were established with images of high and low levels of staining to identify positive staining over all intensity levels within the study. These limits were held constant for the analysis of every section in each study according to Gordon and colleagues [24]*.*

### Western blot analysis

Tissues for western analysis were prepared as described by Carroll and colleagues [25]. Briefly, the dissected anterior cortex tissue was weighed and resuspended in RIPA buffer (50 mM Tris, 140 mM NaCl, 10% NP40, 10% sodium deoxycholate, 10% SDS), with protease inhibitor cocktail (Sigma-Aldrich, St. Louis, MO, USA) and phosphatase inhibitor cocktails I and II (Sigma-Aldrich, St. Louis, MO, USA). Tissue was homogenized with a motorized pestle followed by a brief (10 seconds) sonication pulse. Aliquots were collected at this point for biochemical analysis and were referred to as brain homogenate. Pierce BCA protein assay (ThermoScientific, Waltham, MA, USA) was used to determine protein concentrations. For western analysis, 1 μg protein was loaded for each sample. Antibodies against tau epitopes pS396 and pS199/202 (AT8) were obtained from Anaspec, while H150 antibody was obtain from Santa Cruz Biotechnologies. PHF1 antibody was kindly provided by Dr Peter Davies. A monoclonal antibody against acetylated α-tubulin at Lys40 was used to measure α-tubulin acetylation following treatment (clone 6-11B-1; Sigma-Aldrich, St. Louis, MO, USA). Total α-tubulin levels were measured using monoclonal anti α-tubulin antibody (clone TU-01; ThermoScientific, Waltham, MA, USA).

### Stereology

Methods of design-based (unbiased) stereology were used to quantify hippocampal volumes with assistance from the Stereologer system (Stereologer, St Petersburg, FL, USA). For this study a complete series of sections through the hippocampus was cut at a thickness setting of 50 μm and stained with cresyl violet. From this sample of sections, a systematic random sample of every seventh section was used to estimate the total hippocampal volume using the Cavalieri-point counting method [26].

### Statistical analysis

Statistical analysis and regression plots of studies including both transgenic and nontransgenic mice (behavior) was performed using two-way analysis of variance followed by Fisher’s protected least significant difference (PLSD) *post hoc* means comparison test using StatView version 5.0.1 (SAS Institute Inc, Cary, NC, USA). Measures excluding nontransgenic mice (tau levels) were analyzed by Student’s *t* test.

## Results

**Figure 1:**
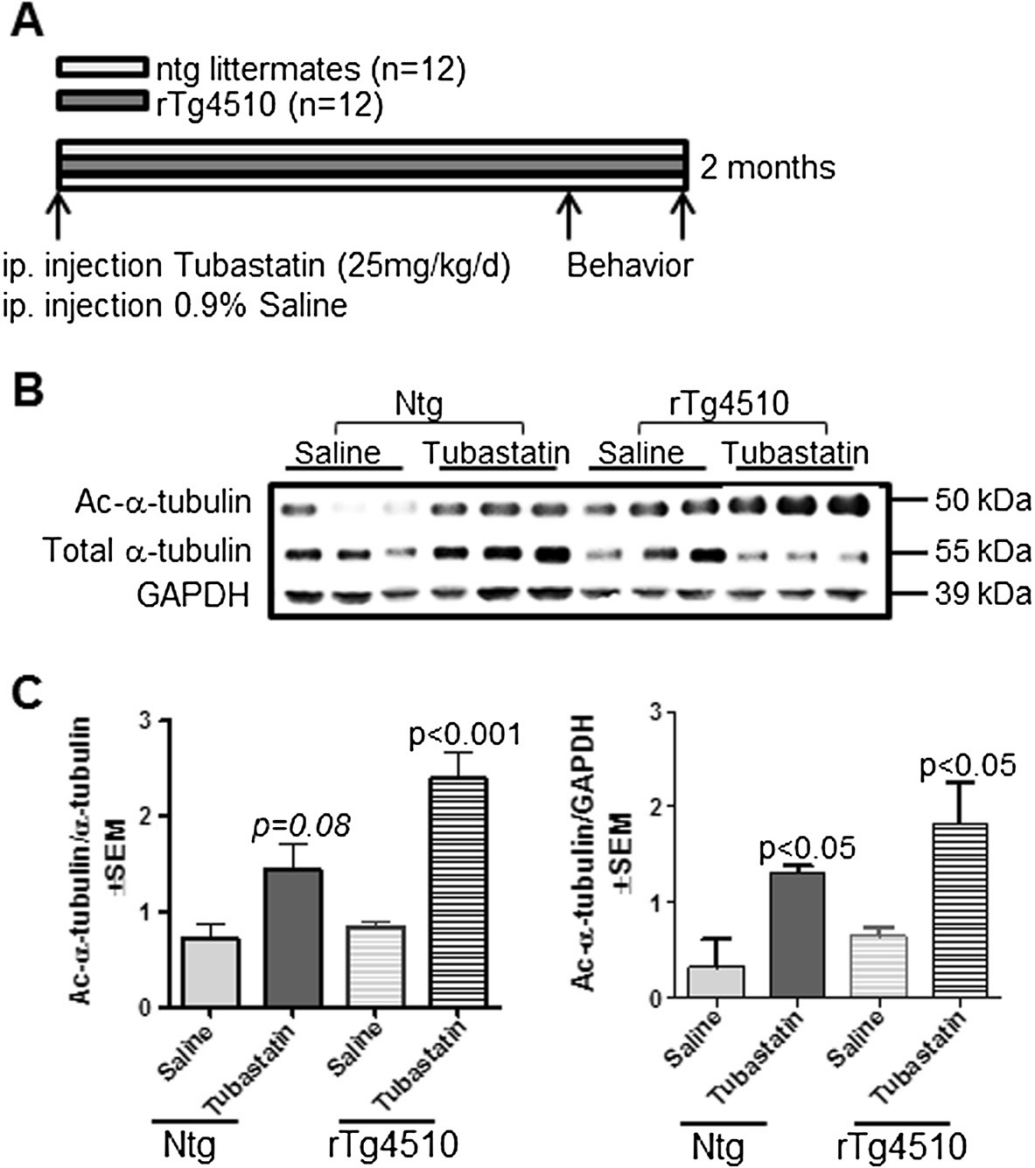
**Schematic presentation of the experimental design of the current study. (A)** Transgenic rTg4510 mice (*n* = 12) and their nontransgenic (ntg) littermates received daily intraperitoneal (i.p.) injections of either 25 mg/kg tubastatin or 0.9% saline. Behavior testing of all animals was performed 2 weeks prior to tissue collection. **(B)** Inhibition of histone deacetylase 6 increased acetylation levels of α-tubulin in vivo. Western blot analysis of brain homogenate from saline and tubastatin-treated ntg and rTg4510 animals were probed for acetylation of α-tubulin at the Lys40 residue and total α-tubulin levels. **(C)** Analysis of acetylated α-tubulin ratio to total α-tubulin and glyceraldehyde 3-phosphate dehydrogenase (GAPDH) by Student’s *t* test confirmed a significant treatment effect of tubastatin in ntg (*P* = 0.08 and *P* < 0.05, respectively) and rTg4510 animals compared with saline-treated littermates (*P* < 0.001 and *P* < 0.05, respectively). All data are presented as mean of ratio ± standard error of the mean (SEM).

To determine the therapeutic effect of HDAC6 inhibition on tau pathology we administered tubastatin, which shows potency and selectivity towards HDAC6 inhibition *in vitro* and *in vivo* (IC50 = 15 nM for HDAC6) [6]. An equal number of 5-month-old rTg4510 mice and nontransgenic littermates were treated with tubastatin or the saline vehicle by daily intraperitoneal injection for 2 months. At week 6 mice were started on a 2-week behavioral test battery, and tissues were collected at the beginning of week 9 (Figure 1A). Measurements of α-tubulin acetylation were used to determine the efficacy of the treatment in modifying the acetylation state of α-tubulin at the Lys40 residue. Western blot analyses revealed a greater than twofold rise in acetylated α-tubulin to total α-tubulin in tubastatin-treated rTg4510 mice, while a 1.5-fold rise was observed in nontransgenic mice as compared with saline-treated littermates (Figure 1B,C). Interestingly, we observed a reduction in the levels of α-tubulin following tubastatin treatment of rTg4510 but not nontransgenic mice (Figure 1B). Data analysis (normalized to glyceraldehyde 3-phosphate dehydrogenase) demonstrated that tubastatin administration significantly induced acetylated α-tubulin levels in both nontransgenic and transgenic animals compared with saline-treated littermates (Figure 1C). These findings are indirectly indicative of the effect of the drug on HDAC6 by enhancing α-tubulin acetylation in the brain.

**Figure 2:**
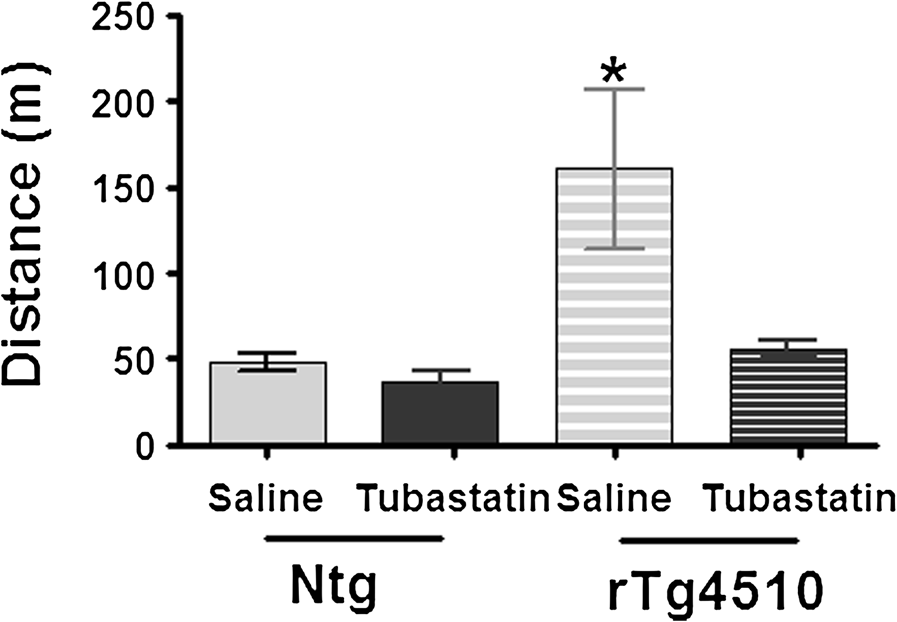
**Rescue of rTg4510 hyperactivity by tubastatin treatment.** Treatment of rTg4510 animals with tubastatin significantly decreased the open field test distance travelled compared with the saline-treated littermates (distance ± standard error of the mean, *n* = 6). Two-way analysis of variance revealed an overall effect of genotype (*P* = 0.03) and of treatment in rTg4510 mice (*P* = 0.03). Ntg, nontransgenic *P < 0.05.

One phenotype we have observed in the rTg4510 line is increased open field activity. This was observed again in these mice by a variety of measures, with more than twice the distance traveled by the saline injected transgenic mice relative to either nontransgenic group. Tubastatin was able to completely reverse this effect of the transgene, and reduced activity to a level comparable with both vehicle and tubastatin-treated nontransgenic mice (Figure 2).

**Figure 3:**
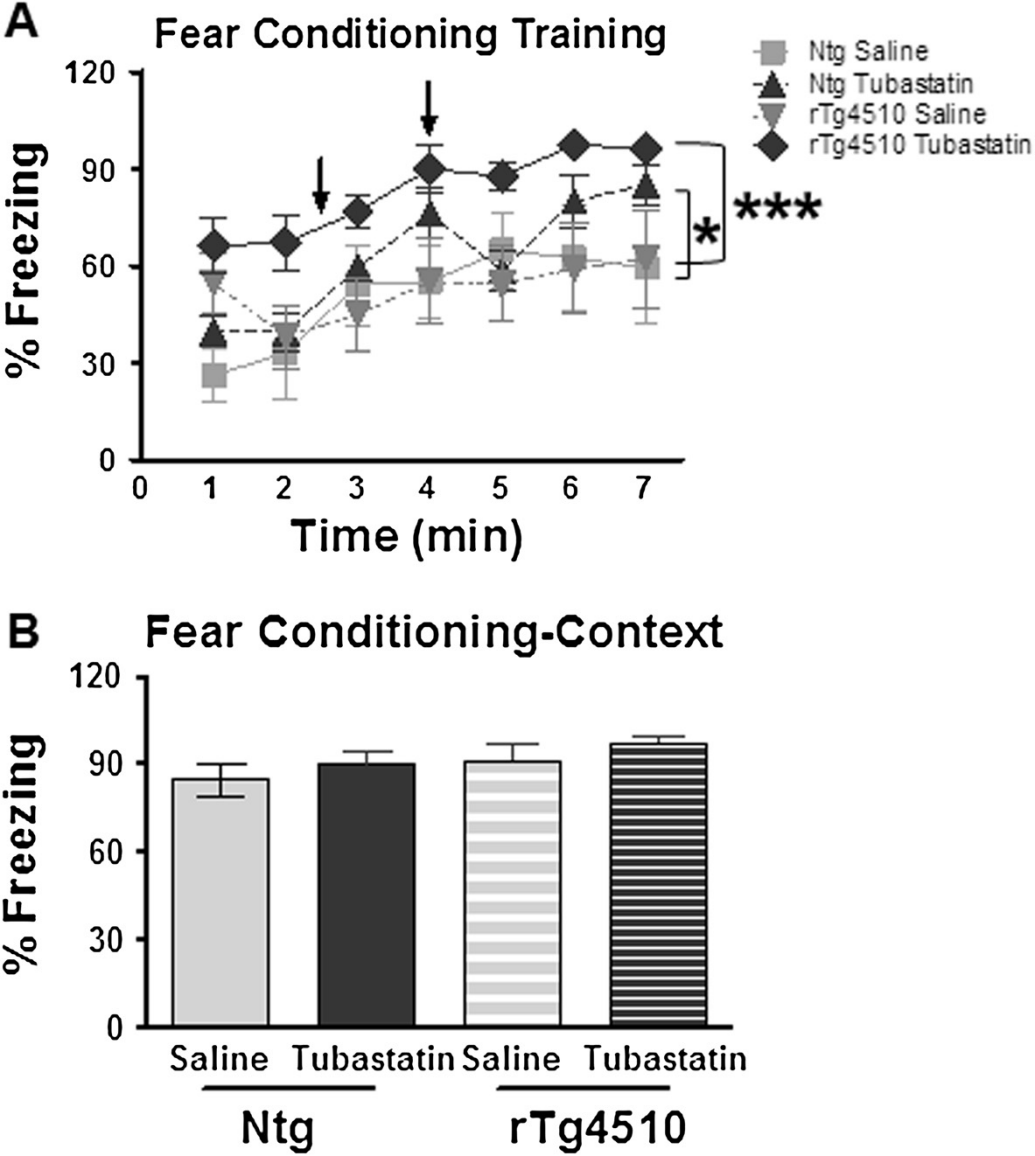
**Increased freezing in tubastatin-treated rTg4510 mice during the acquisition phase of fear conditioning. (A)** Treatment with tubastatin significantly increases the percent freezing ability of the rTg4510 mice compared with the saline-treated littermates (*n* = 6/group) in the acquisition phase of the fear conditioning training paradigm. Two-way analysis of variance followed by Fisher’s PLSD test revealed a significant treatment effect in both transgenic (tg; *P* < 0.0001) and nontransgenic (ntg; *P* < 0.038) animal groups compared with saline-treated mice of the same genotype. **(B)** All mice exhibited high levels of freezing during the retention phase of the test, indicating recall of the context. *P < 0.05, ***P < 0.001.

We also observed an effect of the tubastatin on freezing behavior of the rTg4510 mice in the training trial for fear conditioning (Figure 3A). Here the tubastatin increased freezing in the rTg4510 mice relative to the nontransgenic mice. By the end of the training period, increased freezing was also observed in the vehicle-treated rTg4510 mice. During the contextual recall trial of fear conditioning, all mice exhibited full retention of the context, with close to 100% freezing (Figure 3B). Similar results were seen in the cued retention trial (data not shown).

**Figure 4:**
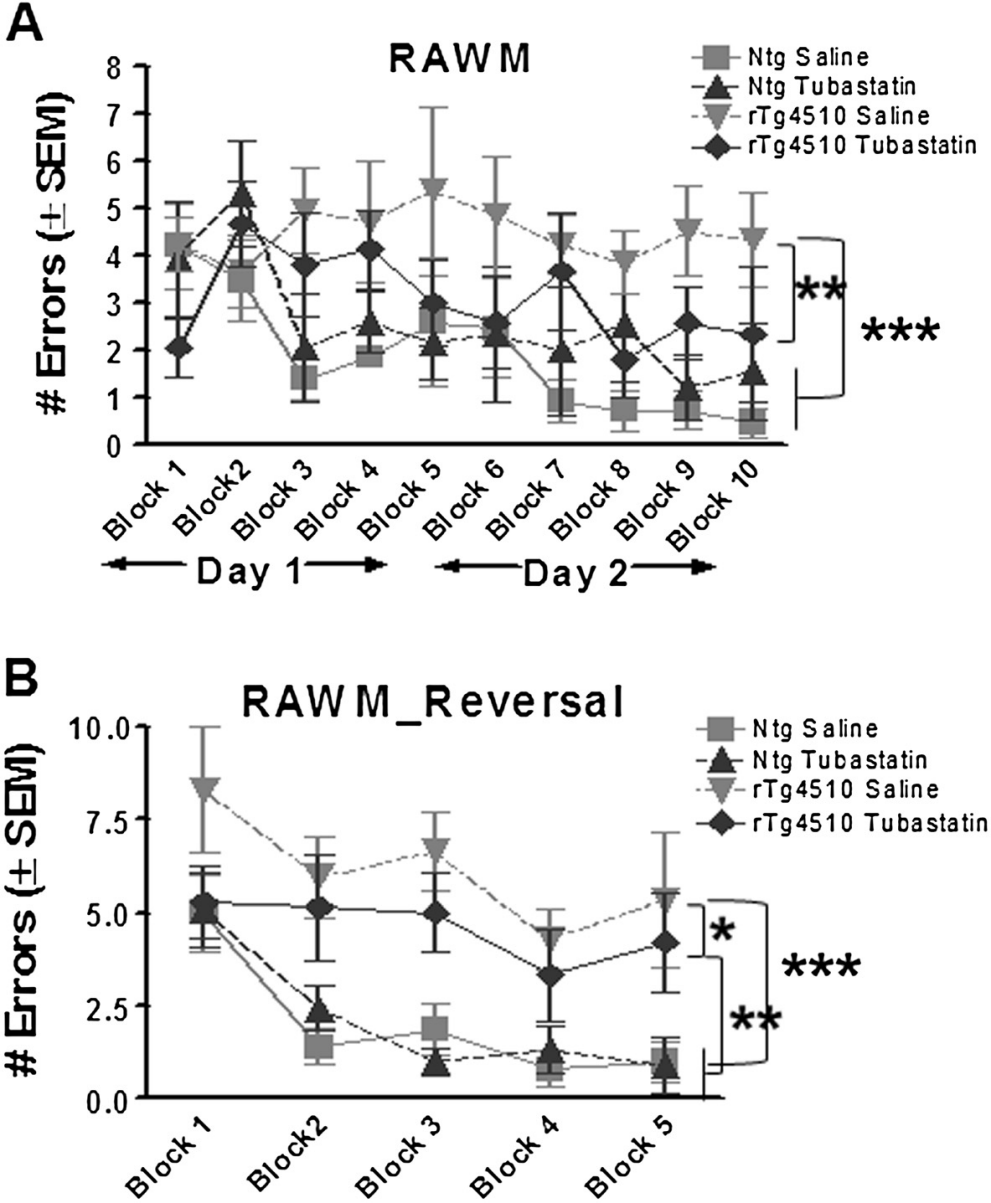
**Rescue of rTg4510 memory impairment by tubastatin treatment.** Number of errors made (average ± standard error of the mean (SEM)) while seeking the hidden platform in the radial arm water maze (RAWM). **(A)** Tubastatin-treated rTg4510 mice performed better in the initial training (5 blocks of three trials) than vehicle-treated littermates (*P* = 0.002) and were not significantly different from the tubastatin-treated nontransgenic (ntg) mice. **(B)** Reversal testing on day 3 (blocks 1 to 5) showed rescue of the memory impairment of rTg4510 mice following histone deacetylase 6 inhibition compared with vehicle-treated littermates (two-way ANOVA and Fisher’s PLSD, *P* < 0.03). No significant difference in either test was detected between vehicle-treated and tubastatin-treated nontransgenic animals (*P* > 0.05). *P < 0.05, **P < 0.01, *** *P* < 0.001.

Mice were then tested for spatial navigation memory using the radial arm water maze task. This task assesses reference memory of hidden platform location within a pool divided into swim alleys. Entries into incorrect arms are scored as errors. Here the vehicle-treated rTg4510 mice failed to demonstrate learning, while both nontransgenic groups achieved the criterion performance of less than one error by the end of the second day of training (Figure 4A). The tubastatin-treated rTg4510 mice showed learning and had significantly fewer errors than the vehicle-treated rTg4510 mice by the end of the task. Moreover, these mice were not significantly different from the nontransgenic mice, although the final mean value did not quite reach the less than one error criterion we have associated with good learning in this task. On the next day the mice were subjected to a reversal task in the radial arm water maze, with the platform moved to a new location. This requires unlearning the prior location and relearning the new platform location. Here the vehicle-treated rTg4510 mice failed to learn the platform location, while both groups of nontransgenic mice rapidly acquired the new platform location. The tubastatin-treated rTg4510 mice were intermediate and significantly better than the vehicle-treated rTg4510 mice, yet statistically worse than the nontransgenic mice (Figure 4B).

**Figure 5:**
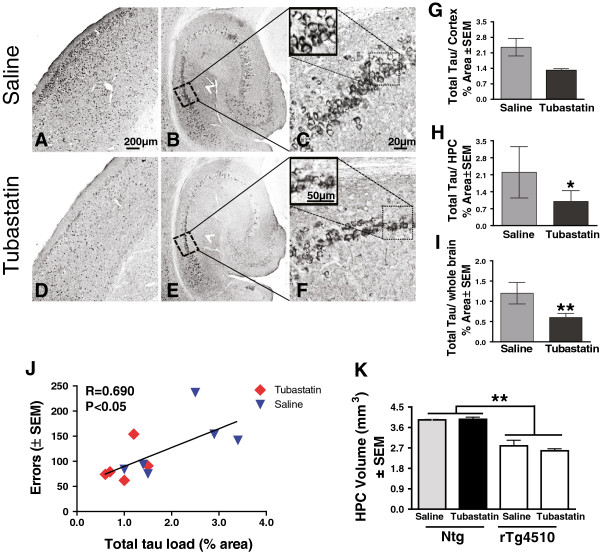
**Tubastatin treatment decreases total human tau levels in the mouse brains with no changes in hippocampal atrophy.** Representative images of brain tissue from **(A, B, C)** saline-treated and **(D, E, F)** tubastatin-treated rTg4510 mice stained for total human tau levels (H150): **(A)**, **(D)** cortex, and **(B)**, **(C)**, **(E)**, **(F)** hippocampus (HPC) at two different magnifications (original magnifications 5×, 10× and 65×). **(B, E)** Arrowhead, neuropil staining. Scale bar: 200 μm, 50 μm and 20 μm. **(G, H, I)** Quantification of immunostaining confirmed reduction of tau levels following treatment: **(G)** cortex (*P* = 0.055), **(H)** HPC (*P < 0.05), and (I) entire hemisphere (**P < 0.001); Student’s *t* test, *n* = 6. Nontransgenic (ntg) mice showed no immunoreactivity against human tau and were thus excluded from the statistical analyses. **(J)** Average number of errors made in the radial arm water maze was plotted against the tau load (H150) measured as the percent positive area in tissue, *n* = 5/6, *P* = 0.0130, *r* = 0.690. **(K)** HPC volume was measured by stereology. The reduced hippocampal volume observed in rTg4510 mice was not affected by the tubastatin treatment (two-way ANOVA, **P < 0.001). Data presented as average +/- SEM.

Tissue sections were stained for a variety of tau isoforms. Stains for total human tau levels with antibody H150 revealed significant reductions in tubastatin-treated rTg4510 mice (nontransgenic mice have no detectable staining in either group). This antibody stains both neurons (darkly) and neuropil (lightly; Figure 5A,B,C,D,E,F, arrowhead). The neuropil staining appears to be specific, as it is not observed in brain regions lacking darkly stained neurons (thalamus, brain stem) or found in nontransgenic mice (not shown). Using the digital scanning microscope, we performed image analysis measures separately in the cerebral cortex, hippocampus and the entire hemisphere. There was a roughly 50% reduction in total tau staining in the tubastatin-treated mice, which was statistically significant in the hippocampus and whole section measurements (Figure 5G,H,I). We also stained sections for a variety of forms of phosphorylated tau, and performed Gallyas silver staining for aggregated tau. None of these markers were modified by the tubastatin treatment (Table 1). Given the positive correlation of tau pathology with cognitive impairment in AD patients [3], we examined how HDAC6 inhibition *in vivo* correlates with behavior and pathophysiological changes measured in treated rTg4510 mice (Figure 5J and Additional file 1). The average number of errors made in the radial arm water maze was plotted against the percent tau load (H150) measured in the tissue of treated rTg4510 animals (Figure 5J). Mice treated with tubastatin significantly reduced the total tau levels, which is positively correlated with the number of errors made during the memory performance test (linear regression, *r* = 0.690, *P* = 0.0130).

**Table 1:** Histone deacetylase 6 inhibition decreases total tau levels but not pathological tau

**Phosphorylation site**	**Saline**	**Tubastatin**	***P *** **value**	**Source**
Ser202/Thr205 (AT8)	0.8 ± 0.1	0.7 ± 0.1	0.46	Ana Spec, EGT group, Fremont, CA, USA
Ser396	0.5 ± 0.1	0. 6 ± 0.1	0.34	Ana Spec, EGT group, Fremont, CA, USA
Ser199/202	0.4 ± 0.1	0.5 ± 0.1	0.25	Ana Spec, EGT group, Fremont, CA, USA
Gallyas stain (NFTs)	1.3 ± 0.2	1.7 ± 0.4	0.21	Fischer scientific, Waltham, MA, USA
Total tau (H150)	2.2 ± 0.4	1.0 ± 0.2	0.03	Santa Cruz Biotechnology, Inc. Dallas, Texas, USA

Mean ± SEM and *P* values are indicated for immunopositive p-tau stain in the rTg4510 saline-treated versus tubastatin-treated animals. Student’s *t* test, *n* = 6. NFT, neurofibrillary tangle.

In addition, tissue stained with cresyl violet was used to estimate the total hippocampal volume as described previously [26]. Stereological analysis showed no effect of tubastatin on the hippocampal volume of treated transgenic mice (Figure 5K), although a clear reduction in the hippocampal volume was observed between rTg4510 mice and nontransgenic littermates. Further, human tau load (percent area) and the number of errors measured in radial arm water maze testing were compared with the hippocampal volume in all subsets of mice (Additional file 1A,B). These data demonstrate that total tau levels and memory performance was negatively correlated with hippocampal volume.

**Figure 6:**
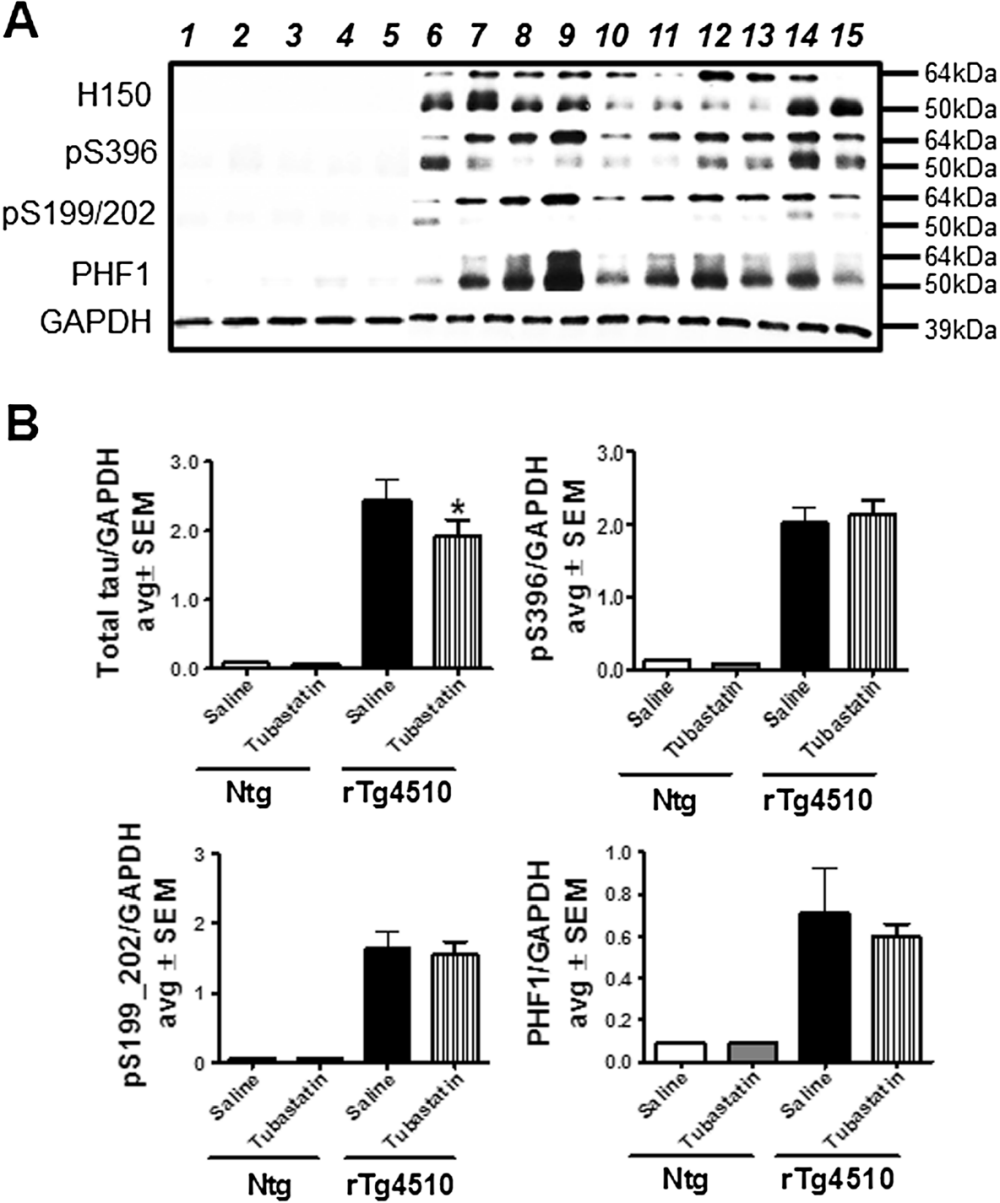
**Tubastatin treatment promotes reduction of total tau levels but not phosphorylated tau. (A)** Levels of total human tau were examined by immunoblotting with H150 antibody (1:1,000 in 1 μg protein) in saline-treated (lanes 1 and 2) or tubastatin-treated (lanes 3 to 5) nontransgenic (ntg) mice, and in saline-treated (lanes 6 to 10) or tubastatin-treated (lanes 11 to 15) rTg4510 mice littermates. Levels of phosphorylated tau present in the brain homogenate were examined using Ser396, Ser199/202 and PHF1 antibodies (1:1,000 dilution). No positive immunoreaction was measured in saline-treated or tubastatin-treated ntg mice. **(B)** Quantification of immunoreactivity normalized to glyceraldehyde 3-phosphate dehydrogenase (GAPDH) revealed a significant decrease in levels of total tau in rTg4510 mice treated with tubastatin compared with saline-treated rTg4510 mice (*P < 0.05). All data are presented as average SEM.

Next, we utilized the contralateral hemisphere and prepared tissue from the anterior cortex for western blot analysis. Once again we found a significant reduction in the total human tau measurement in the rTg4510 mice treated with tubastatin (Figure 6A, lanes 11 to 15) as compared with the saline-treated littermates (Figure 6A,B, lanes 6 to 10). However, the western blot signals for phosphorylated tau at various epitopes were unaffected by the drug treatment, consistent with the histological results. Since tau antibodies recognize anti-human tau, no signal was detected in nontransgenic animals in either group (Figure 6A, lanes 1 to 5).

## Discussion

The primary result of this study is the rescue of the memory impairment found in the rTg4510 mouse line by a 2-month treatment with tubastatin. This reversed both the hyperactivity in these mice and the impaired spatial navigation performance in the initial learning of the radial arm water maze. The nontransgenic mice treated with the drug were no different from the vehicle-treated mice on any of the behavioral tests administered. Rescue of hyperactivity by loss of HDAC6 function has also been recently reported in animal models of amyloid-beta deposition [27]. Interestingly, APPPS1-21 mice crossed with HDAC6^−/−^ mice improved their cognitive deficits without changing the amyloid-beta plaque load. This qualitatively resembles the effects of breeding amyloid-beta depositing mice onto the tau null background (behavioral rescue without impacting amyloid pathology). In our study, the rescue of the spatial navigation should be considered partial, as the mice were deficient compared with nontransgenic mice on the reversal task (albeit improved relative to untreated transgenic mice). One should note that at the initiation of treatment (5 months) rTg4510 mice are already deficient on spatial navigation tasks [12,28]. Moreover, they have already developed considerable pathology, measured histologically [29]. Hence the residual impairment may reflect the impact of pathology that developed prior to the treatment. Several studies have demonstrated that neurodegeneration and associated memory impairments can be rescued by treatment with various HDAC inhibitors treatment (for review see [30]). For instance, treatment of CK-p25 mouse with the nonselective HDAC inhibitor sodium butyrate (class I inhibitor) promoted learning ability and the recovery of long-term memory even after massive neuronal loss [31]. Using a series of elegant experiments, Guan and colleagues demonstrated that chronic treatment of wild-type mice with suberoylanilide hydroxamic acid, a clinically approved inhibitor of class I and class II HDACs, enhanced memory formation more potently than sodium butyrate in contextual fear conditioning training and memory testing paradigm. The authors also provide evidence that gain of HDAC2 function, but not HDAC1, impaired hippocampus-dependent memory formation as well as working memory, implicating HDAC2 in negatively regulating memory formation in mice [32]. Interestingly, levels of HDAC2 were found to be increased by neurotoxic insults *in vitro* in two mouse models of neurodegeneration, and in patients with Alzheimer’s disease [33]. Other studies also demonstrated that treatment of APP/PS1 mice with valproic acid (class I inhibitor [34]) or trichostatin A (class I and class II inhibitor [35]) rescued memory deficits in this model.

These studies support our findings that inhibition of HDAC6 rescued both the hyperactivity and the impaired spatial navigation performance of rTg4510 mice. The literature is less clear as to whether rescue of the behavior is due to reduction of pathology (amyloid-beta or tau). For instance, Ricobaraza and colleagues demonstrated that treatment with sodium 4-phenylbutyrate (class I inhibitor) reversed spatial memory deficits independent of changes in amyloid-beta levels and senile plaque deposits, but did reduce levels of pS202/Thr205 tau in 16-month-old Tg2576 mice [36]. However, chronic administration of sodium 4-phenylbutyrate in 6-month-old Tg2576 mice (prior the onset of disease) prevented memory deficits in these mice and demonstrated decreased levels of amyloid-beta pathology [37]. In our study, behavior improvements following tubastatin treatment were associated with changes in the levels of total tau, not the phosphorylated tau isoforms or the aggregated tau forming argyrophilic deposits detected by Gallyas (argued to be neurofibrillary tangles). This roughly 50% reduction was found throughout the brain both histologically and by western analysis, and is positively correlated with the number of errors made in the radial arm water maze by the mice treated with tubastatin. Superficially, this would imply that the memory impairments on spatial navigation tasks are associated more with the high levels of total tau, rather than with any specific isoform or aggregated tau. This is consistent with the observation of Santacruz and colleagues that inhibition of transgene expression in this mouse led to rescue of the memory impairment, but had no effect on the continued accumulation of abnormally phosphorylated or aggregated tau [12]. This would be consistent with reports that tau aggregation appears to be self-propagating once initiated, and can be spread to adjacent neurons [38,39].

In addition to modification of histones, cytosolic proteins are also substrates for HDACs. Post-translational protein acetylation is important in regulation of several cellular events including microtubule stabilization and intracellular transport [40,41]. In our study, we took advantage of the fact that α-tubulin is one of the main cytosolic substrates of HDAC6. Inhibition of HDAC6 is known to increase acetylation of α-tubulin on Lys40 [40], which was confirmed by our data demonstrating that intraperitoneal administration of tubastatin, a selective inhibitor of HDAC6 (Change back to IC50=15nM) [6], resulted in hyperacetylation of α-tubulin in the brain of the treated mice. Although the mechanism by which tubastatin affects microtubule stability was not investigated here, several studies support this notion [41]. For instance, in Charcot-Marie-Tooth disease it is suggested that the axonopathy is due to destabilization of microtubules and impaired axonal transport [7]. The rescue of this phenotype by tubastatin is suspected to be caused by improved microtubule function associated with increased acetylation. Consistent with this argument, a recent report found that tubastatin restored mitochondrial transport in a cell model of amyloid toxicity [42], while another recent study suggested that inhibition of HDAC6 enzymatic activity by tubastatin increases its direct binding to microtubules and possibly enhanced microtubule stability in human breast cancer cells (MCF-7) [43]. Improved axonal transport may thus be one mechanism by which tubastatin is achieving its benefits in this model.

Another interaction of HDAC6 was reported with tau itself in a cell-based overexpression system [44]. Here aggregates of HDAC6 and tau could be detected with association occurring at the microtubule binding domain of tau (using immunoprecipitation studies). Moreover, these aggregates formed perinuclear structures resembling aggresomes. HDAC6 inhibitors also reduced phosphorylation of tau at position 202/205 (AT180), but not at position 396/404 (PHF-1). Given that the HDAC6 inhibitors used by Ding and colleagues reduced phosphorylated tau but had no apparent effect on total tau, it is uncertain what the relevance of this association might be to the present study. One should also note that Ding and colleagues reported that HDAC6 inhibitors did not block the association between HDAC6 and tau [44]. A similar interaction between tau and HDAC6 was observed by Perez and colleagues using immunoprecipitation [45]. These authors also found in neuronal cultures that tau could inhibit the deacetylation activity of HDAC6 in a dose-dependent manner. They also argued that HDAC6 inhibitors were less effective in increasing tubulin acetylation in the presence of tau. However, given the twofold elevation of tubulin acetylation with tubastatin in the rTg4510 mouse, which expresses many fold more tau than wild-type mice, this action of tau seems to be less important *in vivo* than in cell systems. After we initiated these studies, a second mechanism by which HDAC6 inhibition might affect tauopathy was described. Cook and colleagues showed that, through acetylation of HSP90, HDAC6 regulates the degradation of tau in cell models [8]. When HDAC6 activity is decreased *in vitro*, there is an increased clearance of tau. Elevation of HDAC6 in cells promotes accumulation of tau. These actions appear mediated through inhibition of p23 binding to acetylated HSP90, reducing its affinity for client proteins and enhancing their clearance. HDAC6 inhibition will thus bias the actions of HSP90 towards degradation of its client proteins, reducing the levels of tau.

Our data presented here, with a lowering of total tau, are consistent with this mechanism of action. Notably, these data provide multiple, related mechanisms by which HDAC6 inhibition might have benefits in tauopathies. One interesting observation is the finding that HDAC6 expression is increased by 52% in the Alzheimer’s disease cortex and by 91% in the Alzheimer’s disease hippocampus, when compared with young normal brains [46], as tau pathology progresses from entorhinal to hippocampal to neocortical regions [47]. Although the mechanism of action by which tubastatin reduces total tau and improves the behavior deficits of rTg4510 mouse models was not investigated in this study, our data are the first to demonstrate rescue of behavior phenotype and reduction of total tau levels *in vivo*. Future experiments will exploit the mechanism behind such events.

## Conclusions

Unanswered questions include whether initiating HDAC6 inhibition earlier would not only prevent the behavioral changes, but could also reduce the pathological changes found in the rTg4510 mouse. A second question is whether the benefits would be present in the absence of elevated tau expression, such as after treating rTg4510 mice with doxycycline. Benefits found in the presence of doxycycline would imply that effects in stabilizing microtubules would be a major mechanism, while loss of additional benefit of tubastatin would suggest that the major effect of tubastatin is due to lowering levels of total tau. In any case, these data strongly support development of HDAC6 inhibitors, such as tubastatin, as possible treatments for Alzheimer’s disease and other tauopathies.

## Abbreviations

HDAC: histone deacetylase.

## Competing interests

AK and JK have intellectual property through the University of Illinois regarding the compound tubastatin. Patent number: US2013281484 (A1).

## Authors’ contributions

M-LBS led the study and was involved in all aspects from injections of animals to data analysis, figure generation, data interpretation and manuscript preparation. LB participated in daily injections, behavior testing, stereological procedures, and data acquisition. SBH participated in tissue preparation, biochemical analysis as well as data analysis. BM assisted with immunohistochemical staining, and data acquisition. DCL and KRN participated in data analysis and interpretation, and manuscript revision contributing to its intellectual content. JK and JAB participated in drug design and synthesis. AK supervised and designed drug synthesis as well as contributed to the manuscript’s intellectual content and final approvals. DM and MNG were involved in overall conception, experimental design, data interpretation, manuscript preparation and final approval of the version to be published, as well as securing funding for the project. All authors read, revised and approved the final manuscript. 

## Supplementary Material

Additional file 1 **Is a figure showing hippocampal volume is negatively correlated with total tau levels and memory performance. (A)** Total tau load measured in tissue (H150, % area) was plotted against the hippocampal volume of rTg4510 and nontransgenic (ntg) animals, following treatment (*n* = 5/6, *P* = 0.0001, *r* = 0.714). **(B)** The average number of errors made in the radial arm water maze was plotted against the hippocampal volume of rTg4510 and ntg animals (*n* = 5/6, *P* = 0.001, *r* = 0.657). Click here for file
