# Supplementary figures and images for: Histone deacetylase 6 inhibition improves memory and reduces total tau levels in a mouse model of tau deposition
Source: Alzheimers Res Ther. 2014 Feb 27;6(1):12. doi: 10.1186/alzrt241 (PMC3978441; doi:10.1186/alzrt241)

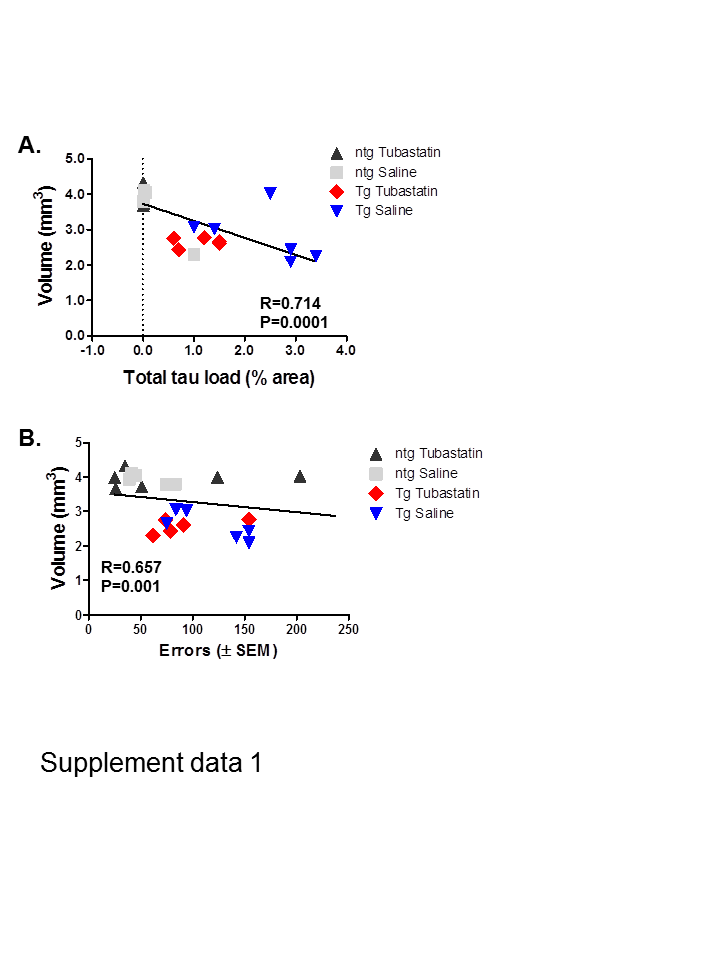

Supplement: Additional file 1 — Is a figure showing hippocampal volume is negatively correlated with total tau levels and memory performance. (A) Total tau load measured in tissue (H150, % area) was plotted against the hippocampal volume of rTg4510 and nontransgenic (ntg) animals, following treatment (n = 5/6, P = 0.0001, r = 0.714). (B) The average number of errors made in the radial arm water maze was plotted against the hippocampal volume of rTg4510 and ntg animals (n = 5/6, P = 0.001, r = 0.657). [file alzrt241-S1.tiff]
